# Examining for Cavum Septum Pellucidum and Ventricular Enlargement in Retired Elite-Level Rugby League Players

**DOI:** 10.3389/fneur.2022.817709

**Published:** 2022-04-06

**Authors:** Peter Stanwell, Grant L. Iverson, Ryan Van Patten, Rudolph J. Castellani, Paul McCrory, Andrew J. Gardner

**Affiliations:** ^1^Priority Research Centre for Stroke and Brain Injury, School of Health Sciences, University of Newcastle, Callaghan, NSW, Australia; ^2^Department of Physical Medicine and Rehabilitation, Harvard Medical School, Boston, MA, United States; ^3^Department of Physical Medicine and Rehabilitation, Spaulding Rehabilitation Hospital, Charlestown, MA, United States; ^4^Spaulding Research Institute, Charlestown, MA, United States; ^5^MassGeneral Hospital for Children Sports Concussion Program, Boston, MA, United States; ^6^Home Base, A Red Sox Foundation and Massachusetts General Hospital Program, Charlestown, MA, United States; ^7^Providence Veterans Administration Medical Center, Providence, RI, United States; ^8^Department of Psychiatry and Human Behavior, Alpert Medical School of Brown University, Providence, RI, United States; ^9^Department of Pathology, Northwestern University Feinberg School of Medicine, Chicago, IL, United States; ^10^The Florey Institute of Neuroscience and Mental Health, Parkville, VIC, Australia; ^11^Priority Research Centre for Stroke and Brain Injury, School of Medicine and Public Health, The University of Newcastle, Callaghan, NSW, Australia; ^12^Hunter Medical Research Institute, New Lambton Heights, NSW, Australia

**Keywords:** concussion, traumatic brain injury, magnetic resonance imaging (MRI), biomarkers, cavum septum pellucidum, cognition, rugby

## Abstract

**Objective:**

A cavum septum pellucidum (CSP) has been reported as a visible brain anomaly in normal individuals as well in some former combat and collision sport athletes. The appearance of CSP with fenestrations and ventricular enlargement are considered associated features of the neuropathological diagnosis of chronic traumatic encephalopathy. The current study examined CSP anatomic features and lateral ventricle size in retired elite rugby league players and controls.

**Methods:**

Forty-one retired rugby league players and 41 healthy community controls, similar in age and education, underwent structural MRI scans. CSP grade, CSP length, corpus callosum septal length, and Evans' ratio (for lateral ventricle size) were rated by two of the current study authors. All participants also self-reported concussion exposure histories, depressive symptoms, daytime sleepiness, and impulsivity. They completed a neuropsychological test battery assessing premorbid intellectual functioning, attention, processing speed, language, visuospatial skills, memory, and aspects of executive functioning.

**Results:**

The two raters had high agreement for CSP grade (Cohen's κ = 0.80), CSP length [intraclass correlation (ICC) = 0.99], corpus callosum septal length (ICC = 0.73), the CSP/septal ratio (ICC = 0.99), and the Evans' ratio (ICC = 0.75). Twenty-five retired players (61.0%) had an abnormal CSP compared to 17 controls [41.5%; $\chi_{\left(1,\;82\right)}^{2}$ = 3.12, *p* = 0.08, odds ratio = 2.21]. The CSP/septal ratio was larger for retired players than for the controls. The Evans' ratio did not differ between the two groups. In the retired rugby league players (*n* = 41), those with normal (*n* = 16) and abnormal (*n* = 25) CSP grades did not differ across age, age of first exposure to collision sport, years of sport exposure, concussion history, or 23 clinical and cognitive variables.

**Conclusion:**

This study revealed a difference in the size of the CSP between retired professional rugby league players and controls. There was no significant difference in the size of the ventricles between the two groups. There were no significant differences between those with vs. without an abnormal CSP on age of first exposure to rugby league, years of exposure to repetitive neurotrauma, number of lifetime concussions, depression, impulsivity, perceived cognitive decline, or on any neuropsychological test.

## Introduction

The septum pellucidum is a thin midline structure that divides the lateral ventricles; it consists of two leaves of neuroglial tissue connected via the median forebrain bundle to the hippocampus and via the fornix to the limbic system. The septum develops from the commissural plate, along with the corpus callosum, anterior commissure, and hippocampal commissure (1). Rostrally, the septum forms the medial wall of the bodies of the lateral ventricles, it is bounded posteriorly by the fornix with the rostral and superior limits formed by the corpus callosum. The septum is part of the limbic system, containing both gray matter and white matter tracts that link several structures including the hypothalamus, hippocampus, amygdala, habenula, and brainstem reticular formation (2).

During normal fetal development, the two leaves of the septum are separated and form a cavity referred to as the cavum septum pellucidum (CSP), fusing back together around 36 weeks gestation so that only about one-third of term infants maintain the fetal state (3). While the presence of a CSP is considered to be a *normal* neurodevelopmental anatomical variation in some people (4, 5), it is also considered to be a neurodevelopmental abnormality in people with bipolar disorder (6–8), posttraumatic stress disorder (9, 10), schizophrenia spectrum disorders (6, 8, 11, 12), and telomere biology disorders (13). A substantial percentage of the general population has a CSP, and the reported percentages vary considerably based on the method used to identify it. Pathologically, CSP was noted in 20.3% of 1,032 consecutive lifespan brains at autopsy (14), while Bogdanoff and Natter (15) reported an incidence of only 0.73% in a study of 1,914 adult patients relying on early-generation computed tomography. In contrast, Tsutsumi et al. (16) detected CSP on magnetic resonance imaging (MRI) in 44% (31/71 participants), while Born et al. (17) detected CSP in 80% of children, 68% of young adults, and 72% of older adults (108/151 participants). Although the presence of CSP is frequent in the general population, the presence of an enlarged CSP (>6 mm in length) is infrequent, occurring in 8.7% of children, 11.1% young adults, and 3.0% elderly subjects in one study (17).

### CSP in Combat and Collision Sport Athletes

The presence of a CSP was a common finding in historical studies undertaken in high exposure former boxers who have undergone pneumoencephalography (18–20) and in several post-mortem studies of retired boxers (21, 22). One of the first reports of the presence of a septal cavum at autopsy was described by Grahmann and Ule, who noted that the brain of the former boxer was considerably atrophied, that the lateral ventricles were enlarged, and that a sizable septal cavum was present (23). Additionally, Corsellis observed the fornix was almost totally detached from the inferior surface of the corpus callosum, presence of flattened forniceal bodies splayed over the thalamus, markedly thinned corpus callosum, and that the lateral and third ventricles were commonly enlarged at autopsy (21). The cause of enlarged CSP in these boxers is unclear but it has been hypothesized to be the result of repeated acceleration-deceleration forces resulting in septal fenestrations (18, 19) and repeated sudden increases in intraventricular pressure that forced cerebrospinal fluid (CSF) through defects in the septal leaflets (20, 24).

An enlarged CSP has been reported in contemporary neuroimaging (MRI) studies of boxers (25), active and retired mixed martial arts fighters (26), and retired professional football players (27–29). Additionally, Wilde et al. identified expansion of the frontal horns of the lateral ventricles in a mixed cohort of active and retired boxers using the Evans' index (i.e., the ratio of the maximal transverse diameter of the frontal horns of the lateral ventricles to the maximal internal diameter of the cranium). However, researchers have not found an association between CSP and number of prior concussions (28), years of exposure to collision sports (28), or most clinical outcome variables (26, 29). In one study of 17 retired professional football players and 17 age/sex-matched controls (with no documented TBI histories), the CSP was of a significantly higher grade and longer in players than controls. However, in the group of retired players, neither CSP length (anterior to posterior) nor CSP grade were associated with age, history of concussion with loss of consciousness, years of football exposure (total or professional), years since retirement from football, or Mini-Mental State Examination scores (28). In a larger study of 72 symptomatic retired professional football players and 14 former professional non-contact sport controls, the CSP was present more often and was significantly longer in the retired football players compared to the controls. Next, the retired football players were grouped into CSP ≥ 6 mm (*n* = 35) and CSP ≤ 2 mm (*n* = 23) (29). They were then compared across 24 cognitive and behavioral variables measuring impulsivity, depression, aggression, hopelessness, premorbid intellectual functioning, attention, processing speed, language, visuospatial skills, learning and memory, and aspects of executive functioning. In two of the 24 comparisons, former football players in the CSP ≥ 6 mm group performed significantly worse than the players in the CSP ≤ 2 mm group (immediate recall on a list learning test and the total score on a test of single word reading). The other 22 comparisons were non-significant.

With respect to research in professional fighters, Lee et al. (26) examined clinical and cognitive variables in 476 active and retired professional fighters and 63 controls. The fighters were significantly more likely to have a measurable CSP than were the controls, and the cavum vergae (the posterior extension of the CSP) was also measured. The combined CSP and cavum vergae lengths did not differ between the two groups. In the professional fighters, longer combined CSP and cavum vergae lengths were associated with lower scores on a processing speed composite score and on a psychomotor speed composite score. CSP length alone was not statistically related to 13 variables measuring depression, impulsivity, daytime sleepiness, attention, motor control, reaction time, processing speed, psychomotor speed, verbal memory, and aspects of executive functioning.

Studies to date have focused on boxers, mixed martial artists, and former American football players. Research is needed on former high exposure athletes from other contact and collision sports. Rugby league is a high-intensity collision sport involving a large number of tackles per game (30, 31), frequent blows to the head (32–34), and a fairly high rate of concussions (up to 40 concussions per 1,000 player hours) (35). For the past several years, a study of the brain health of retired elite level rugby league players from Australia has been underway, and these studies have revealed differences in brain macrostructure (36), neurochemistry (37), white matter microstructure (38), and functional connectivity (39).

### Purpose and Hypotheses

The purpose of the current study is to determine whether retired elite level rugby league players show increased evidence of CSP and expansion of the lateral ventricles compared to controls. We had two primary hypotheses. First, we hypothesized that a greater proportion of retired rugby league players would have an abnormal CSP, defined as a grade 2 or higher, than control participants. For this study, we used a grading system (0–4) for CSP, with grade 0 representing no evidence of CSP, grade 1 representing “questionable” or “equivocal” CSP, and grades 2–4 represent increasing size of CSP. Second, we hypothesized that the retired rugby league players would have larger lateral ventricles, as measured by the Evans index (40), than controls. Finally, based on largely null findings from prior studies (26, 28, 29) we anticipated that there would be no significant associations between CSP length or grade and neuropsychological or mental health outcome measures in the retired rugby league players.

## Methods

### Participants

Participants were recruited as part of the Retired Professional Rugby League Players Brain Health Research Program. All were retired male elite-level rugby league players who competed at the first grade level in the New South Wales Rugby League (NSWRL, 1908–1994), Australian Rugby League (1995–1997), Super League (1997), and/or National Rugby League (1998–present). Two methods of recruitment were used. First, club “old boys” (alumni) networks distributed study information to their members. Second, direct referrals were received from two sources: (i) the National Rugby League, and (ii) the Men of League Foundation, who also distributed study information to their members and provided the option to self-refer to the research program. This method of recruitment and selection might have resulted in some retired rugby league players with cognitive dysfunction and/or severe depression being less willing or able to participate in the study—although there could also be some degree of recruitment bias in the other direction, whereby those who are the healthiest, highest functioning, and who have busy personal and professional lives, might have been less likely to participate.

Of 46 retired players with data available for the current study, enrolled during the first 5 years of this study, five were excluded because of motion artifacts that precluded the analysis of CSP, resulting in a final sample size of 41. All participants underwent a clinical interview to collect demographic information, medical history, and concussion history. The clinical interview included collecting self-reported information pertaining to the number of concussions the retired athletes sustained during their career. All participants were provided with a definition of concussion based on the 2012 Zurich consensus statement on Concussion in Sport (41).

Healthy community control participants, similar in age and education, were recruited through a research participant registry developed and managed by the local medical research institute. The registry was established to support research recruitment *via* a central database of people who are interested in contributing to medical research and offers access to a wide cross-section of the population. All controls reported no previous history of concussion or any history of participation in any organized contact, collision, or combat sports. Exclusion criteria for both groups included any medical history of neurosurgery or medical contraindications to magnetic resonance imaging (MRI).

### MRI Data Acquisition

MRI data acquisition was performed on a Siemens 3T scanner, MAGNETOM Skyra (Siemens AG, Healthcare Sector, Erlangen, Germany), using a commercially available RF receive-only 20-channel brain array head coil (Siemens Medical Systems, Erlangen, Germany). The body coil was used for RF transmission. Foam cushions were used to minimize head motion. Structural 3D T_1_-weighted Magnetization Prepared Rapid Gradient Echo (MP-RAGE) images of the whole brain were acquired using the following sequence parameters: TR/TE/TI: 2,200/3.5/1,100 ms; flip angle: 7°; 176 sagittal slices with 1 mm thickness, FOV_AP×*FH*_: 256 × 256 mm^2^, acquisition matrix: 256 × 256 pixels.

#### Post Processing

Image datasets were visually inspected for distortion, motion, and image artifacts, and those free of artifacts were transferred to a separate workstation for further analysis (5 were excluded, as noted above). Following image transfer an additional para-coronal image series was created perpendicular to the axis between the genu and splenium of the corpus callosum to optimize identification, measurement of the length, and grade of CSP along the entire length of the septum pellucidum. These landmarks were chosen because the development of the corpus callosum is closely associated with the development of the septum pellucidum (1). Images were reformatted with 1 mm slice thickness and no slice gap.

#### Rating and Characterization of CSP

Characterization of the septum and CSP used a combination of methods including (1) CSP grade, (2) CSP length, and (3) septal length (the longest intraventricular distance from genu to splenium of the corpus callosum). CSP was defined as cerebrospinal fluid (CSF) being present between the two leaflets of the septum pellucidum on paracoronal T1-weighted images. Evaluation of CSP presence and grade was rated using a previously published ordinal scale, and was determined from the paracoronal slice with greatest evidence of a cavity (28, 42, 43). The rating grades assigned were Grade 0: the septum appeared crisp without any evidence of cyst (absent); Grade 1: the septum demonstrated slight interior hypointensity that was not clearly CSF signal intensity (septum unclear, questionable). Grades 2–4 demonstrated clear evidence of CSF signal between the separated leaflets of the septum pellucidum. The degree of separation between the leaves of the septum was then used to assign a grade of 2–4. Grade 2 (mild): CSP is not wider than the septum itself, Grade 3 (moderate): the CSP is wider than the septum but less than half the intraventricular width, and Grade 4 (severe): CSP is greater than half the intraventricular width (28). The presence of a CSP was defined as a rating of 2 or greater.

The length of the cavum was determined by summing the number of 1 mm paracoronal slices where CSP was visible to calculate the overall length of the CSP (28, 29, 42). To account for potential bias of anatomic variation of individual head size and ventricular enlargement, the longest distance from the genu to splenium of the corpus callosum (“septal length”) was assessed in the midsagittal plane using the workstation measurement tool. To adjust for individual septal length, the ratio of CSP length to septal length was calculated (i.e., CSP length/septal length).

#### Evans' Ratio for Ventricle Size

Evans' ratio is a radiological tool for the reliable assessment of ventricular enlargement (44). Originally based on pneumoencephalographic findings, subsequent studies have confirmed its reliability using more advanced neuroimaging techniques (45, 46), and favorable comparison to ventricle volumetric analysis for assessment of ventriculomegaly (46). Evans' ratio was determined by calculating the ratio of the maximal transverse diameter of the frontal horns of the lateral ventricles to the maximal internal diameter of the cranium. International guidelines define ventricular enlargement as Evans' ratio >0.3 (45, 46), though the guidelines do not specify whether the enlargement relates to brain atrophy or disturbance of CSF dynamics.

#### Exposure Variables and Clinical Measures

Participants self-reported their concussion histories. They were provided with the definition of concussion from the consensus statement on concussion in sport, 2012 (41), and they asked questions to clarify the definition, if necessary. Participants also reported their histories of sport and non-sport concussions; lifetime concussions were the sum of these values. In addition, they reported both years playing professional rugby league and total years playing rugby league (at any level).

Clinical measures were self-reported health questionnaires and tests of cognitive abilities. Questionnaires included the Informant Questionnaire on Cognitive Decline in the Elderly, self-report version (IQCODE-Self) (47), the Depression, Anxiety, Stress Scale 21-item (DASS-21) (48), the Epworth Sleepiness Scale (ESS) (49), and the Barrett Impulsivity Scale (BIS) (50). Premorbid intellectual functioning was estimated with the Advanced Clinical Solutions (ACS) Test of Premorbid Functioning (ToPF) word reading standard score (M: 100; SD: 15) (51). Additional cognitive tests were as follows: Mitrushina et al. (52) meta-norms converted raw data to standard scores for the Rey Auditory Verbal Learning Test (RAVLT) Trials 1–5 (53), Rey Complex Figure Test (RCFT) Immediate Recall and Delayed Recall (54), Trail Making Test (TMT) A and B (55), the Controlled Oral Word Association Test (COWAT) FAS and Animal Fluency (56), and Stroop condition 3 – Inhibition (57). The Wechsler Adult Intelligence Scale, 4th Edition (WAIS-IV) (58). Australian and New Zealand norms converted the raw data to standard scores for Digit Span (Backwards and Sequencing, separately), Symbol Search, and Coding. These tests, excluding the ToPF, provided twelve age-corrected scores, which were converted to T scores. The means of the twelve scores were calculated for each participant to produce a cognitive composite score.

### Statistical Analyses

Multiple continuous variables were non-normally distributed, so non-parametric statistics were used where appropriate. Wilcoxon signed rank test examined differences in related variables (Rater 1 vs. Rater 2). Mann Whitney U examined differences across independent groups (retired rugby league players vs. controls or normal vs. abnormal CSP). For comparisons across categorical variables, chi square tests were conducted.

Interrater reliability was assessed by Spearman correlations, intraclass correlations (ICCs), and non-parametric statistics for continuous variables, and by percent agreement and Cohen's kappa for binary variables. We selected the single-rating, absolute-agreement, 2-way random effects model for ICCs because (i) we used the same raters (authors PS and AG) for all ratings, (ii) we want to generalize to other raters, (iii) we are interested in absolute agreement rather than consistency, and (iv) we are interested in ratings from single raters in the real world rather than mean ratings (59). We present the results from both raters to illustrate the consistency and reproducibility of the findings.

Retired rugby league players were compared to controls with respect to CSP length, septal length, their ratio, and Evans' ratio, similar to prior studies in combat and collision sport athletes (24, 26, 28, 29, 60). The primary analyses of interest were to compare the two groups on the presence of an abnormal CSP, the size of the CSP (i.e., the ratio of the CSP length and septal length), and the size of their ventricles. For examination of potential group differences in clinical and concussion exposure variables, the retired rugby league players group was categorized into normal (0–1; *n* = 16) vs. abnormal (2–4; *n* = 25) CSP rating, and non-parametric tests were conducted. These subgroup analyses were meant to determine if statistically significant results from prior studies would replicate in the present sample. These findings include associations between larger CSPs and worse performance on one test of list learning (immediate recall) and one test of single word reading in retired professional football players (29), and relationships between larger combined CSP/cavum vergae and worse performance on a composite measure of processing speed and a composite measure of psychomotor speed in professional fighters (26). Exploratory clinical-anatomical correlations were also examined within the retired rugby league players using Spearman correlations.

Given the large number of comparisons conducted for the clinical outcome measures, we considered an adjustment for alpha inflation (e.g., Bonferroni or false discovery rate). However, given our relatively small sample size of retired rugby league players (*n* = 41), together with null findings from prior studies on this topic, we elected to minimize the chance of a false negative error by setting statistical significance at *p* < 0.05. That is, in weighing Type I and Type II error, we chose to maximize our ability to detect possible true associations that may be present between CSP length or grade and clinical outcomes, at the expense of a meaningful risk for false positive errors.

## Results

### Examining the Presence of a CSP

**Figure 1:**
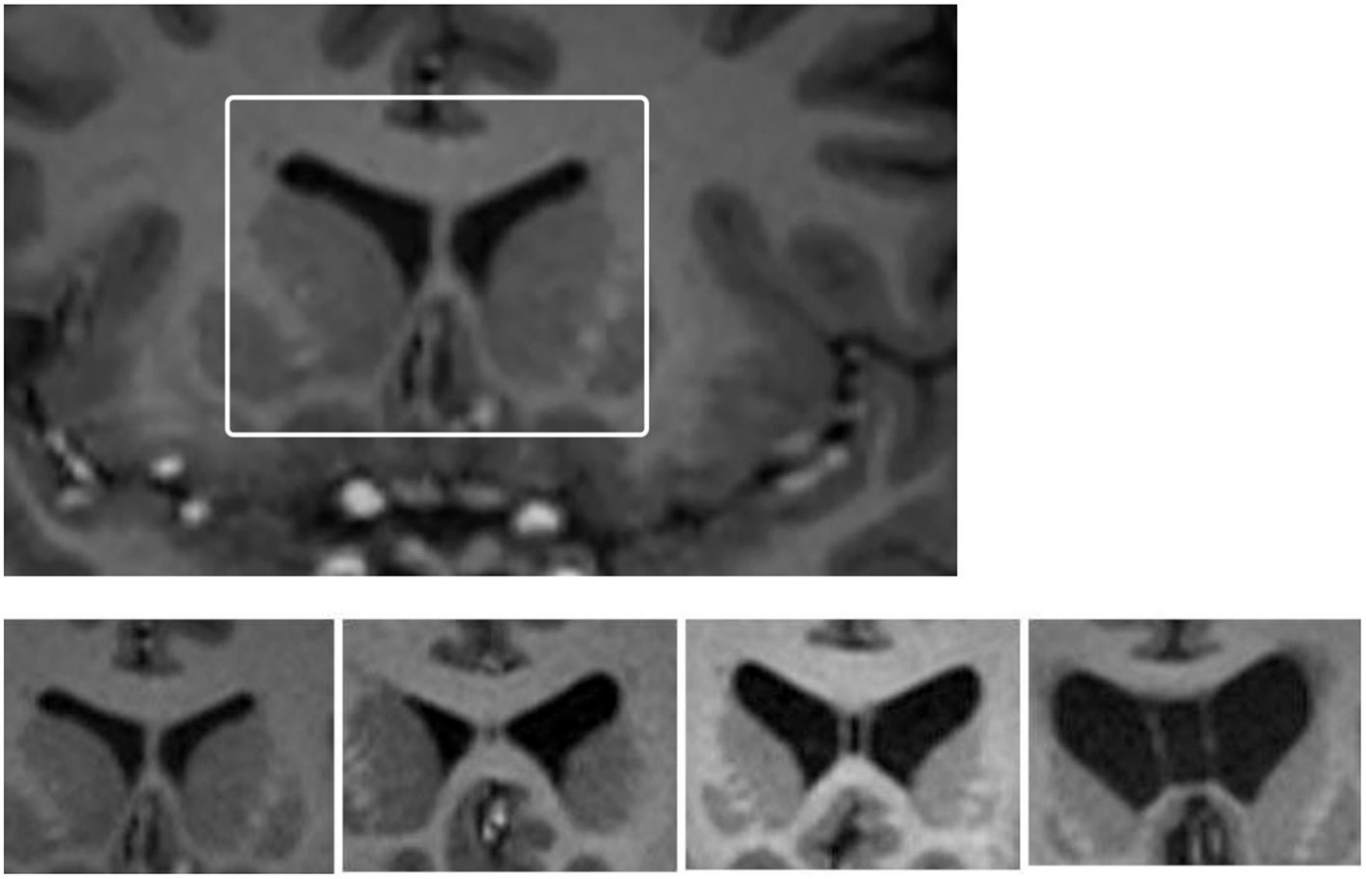
Images of grades of CSP. Cavum septum pellucidum (CSP) grading system for grades encountered in this study. Top: White box depicts region that was enlarged to undertake CSP grading for each participant. Bottom: from left to right the four-image panel represents CSP grades of 0 (absent), 1 (slight), 2 (mild), and 3 (moderate). There were no grade 4 (severe) cases in this study.

CSP grade (see Figure 1) was determined by two raters who were blinded to clinical information at the time of rating (Rater 1, PS and Rater 2, AG). Of the 82 participants, Rater 1 identified 40 (48.8%) as having an abnormal CSP (grade of 2 or greater) and Rater 2 identified 36 (43.9%) as having an abnormal CSP. Overall, they had a strong rate of agreement (61) [Percent Agreement = 90.2% (74/82); Cohen's κ = 0.80 (SE = 0.07; *p* < 0.001)].

The final grade used in the analysis was determined based on re-examination and discussion of cases, to form a consensus grading. No participant in either group had a “severe” CSP, graded as a 4/4. There were 1/41 control participants (2.4%) and 4/41 retired players (9.8%) with a Grade 3 (“moderate”) CSP. There were 25 retired players (61.0%) who had an abnormal CSP, defined as Grade 2 or 3, compared to 17 controls [41.5%; $\chi_{\left(1,\;82\right)}^{2}$ = 3.12, *p* = 0.08, odds ratio (OR) = 2.21, 95% confidence interval (CI) = 0.91–5.33].

### Examining the Length of CSP and the Septal Length

Length of CSP, septal length, and their ratio were examined in the full sample (*n* = 82). CSP length was compared for the two raters. The Spearman correlation between the two raters' scores was *r* = 0.98 (*p* < 0.001), the ICC was 0.99 (95% CI = 0.990–0.996; *p* < 0.001), and there was no difference in their average ratings (Rater 1 Mdn = 3.00; Rater 2 Mdn = 3.00; T = 400, *p* = 0.14). For septal length, the Spearman correlation between the two raters was *r* = 0.80 (*p* < 0.001), and the ICC was 0.73 (95% CI = 0.43–0.86; *p* < 0.001). There was a statistically significant difference between the two raters on the average septal length (Rater 1 Mdn = 5.36; Rater 2 Mdn = 5.05; T = 574, *p* < 0.001). The ratios of the CSP length and the septal length were highly correlated between the two raters (Spearman *r* = 0.97, *p* < 0.001; ICC = 0.99, 95% CI = 0.98–0.99, *p* < 0.001). The ratios differed between the two raters (Rater 1 Mdn = 0.55; Rater 2 Mdn = 0.61; T = 1,627, *p* < 0.001).

Descriptive statistics and group comparisons (retired rugby league players vs. controls) for the CSP length, septal length, their ratio, by rater, are presented in Table 1. Results of all group comparisons were similar across the two raters. The length of the CSP and the CSP/septal ratio were greater for the retired players than for the controls, while septal length did not differ across the two groups. Importantly, the CSP and the CSP/septal ratio are redundant variables, so only the CSP/septal ratio should be interpreted (i.e., their Spearman correlation is *r* = 0.991 in the control participants and *r* = 0.995 in the retired players).

**Table 1:** Group comparisons on CSP and ventricle size.

	**Retired rugby league players**				**Controls (*****n*** **= 41)**						
	**(*****n*** **= 41)**										
	**M**	**Md**	**SD**	**IQR**	**M**	**Md**	**SD**	**IQR**	**U**	***p***	***r***
**Rater 1 (PS)**											
Length of CSP^*^	6.88	5.00	7.42	2.00–9.50	3.61	2.00	8.62	0.00–3.50	**1,178.00**	**0.002**	**0.35**
Septal length	5.37	5.46	0.50	5.06–5.72	5.25	5.26	0.46	4.92–5.57	986.00	0.18	0.15
CSP/septal ratio^*^	1.29	1.02	1.35	0.35–1.78	0.68	0.43	1.51	0.00–0.65	**1,162.00**	**0.003**	**0.33**
Evans' ratio	0.27	0.27	0.02	0.25–0.29	0.27	0.27	0.02	0.25–0.28	901.00	0.58	0.06
**Rater 2 (AG)**											
Length of CSP^*^	6.76	5.00	7.10	2.00–10.00	4.05	3.00	8.65	0.00–4.00	**1,124.50**	**0.008**	**0.29**
Septal length	5.14	5.29	0.61	4.71–5.65	5.02	5.01	0.54	4.66–5.37	941.00	0.35	0.10
CSP/septal ratio^*^	1.32	1.01	1.35	0.39–1.96	0.79	0.50	1.49	0.00–0.85	**1,114.00**	**0.01**	**0.28**
Evans' ratio	0.26	0.27	0.03	0.24–0.28	0.26	0.26	0.02	0.24–0.28	884.00	0.69	0.04

* *The length of the CSP and the CSP/Septal Ratios are redundant variables; their Spearman correlations are r = 0.991 (Rater 1) in the control participants and r = 0.995 (Rater 1) in the retired players. Only the CSP/Septal Ratio should be interpreted. In the text, we refer to this significant difference between CSP/Septal Ratio as revealing a “larger” CSP in the retired players*.

*CSP, cavum septum pellucidum; IQR, interquartile range; Md, median; M, mean; SD, standard deviation; U, Mann Whitney U statistic; p, probability of a Type I error; r, correlation coefficient. Effect sizes are interpreted as 0.1 = small, 0.3 = moderate, 0.5 = large*.

### Examining Ventricle Size

Evans' ratios were computed by Rater 1 and Rater 2. The correlation between the two ratings was high (Spearman *r* = 0.85, *p* < 0.001; ICC = 0.75, 95% CI = 0.53–0.86, *p* < 0.001). There was a statistically significant difference between the two raters on the Evans' ratio (Rater 1 Mdn = 0.27; Rater 2 Mdn = 0.26; T=564, *p* < 0.001).

The Spearman correlations between the Evans' ratio and CSP/septal ratio in the control group were *r* = −0.39, *p* = 0.01 (Rater 1) and *r* = −0.26, *p* = 0.11 (Rater 2). Within the control group, the distribution of Evans' ratio scores was significantly different between those with an abnormal CSP (*n* = 17) and those with no CSP (*n* = 24; U = 105.0, *p* = 0.009 Rater 1; U = 123.0, *p* = 0.032 Rater 2), with those with an abnormal CSP having a significantly *smaller* Evans' ratio. The Spearman correlations between the Evans' ratio and age were *r* = 0.44, *p* = 0.004 (Rater 1) and *r* = 0.39, *p* = 0.01 (Rater 2) for the controls.

The Spearman correlations between the Evans' ratio and CSP/septal ratio in the retired players group were *r* = −0.16, *p* = 0.33 (Rater 1) and *r* = −0.19, *p* = 0.24 (Rater 2). Within the retired players group, the distribution of Evans' ratio scores was not significantly different between those with an abnormal CSP (*n* = 25) and those with no CSP (*n* = 16; U = 192.0, *p* = 0.843 Rater 1; U = 189.0, *p* = 0.781 Rater 2). The Spearman correlations between the Evans' ratio and age were *r* = −0.48, *p* = 0.002 (Rater 1) and *r* = 0.29, *p* = 0.07 (Rater 2) for the retired players. The Evans' ratio did not significantly differ between the retired players and the controls (Table 1).

### Examining Clinical Measures and Concussion Exposure Variables

Within the group of retired Rugby League players, those with normal (*n* = 16) and abnormal (*n* = 25) CSP grades were compared across age, concussion exposure, and clinical variables. Clinical variables included measures of depression, daytime sleepiness, impulsivity, estimated premorbid intelligence (i.e., word reading), attention, processing speed, language, visuospatial skills, memory, and aspects of executive functioning. None of the 23 group comparisons were statistically significant. Most effect sizes were small or negligible (Table 2).

**Table 2:** Clinical measures and concussion exposure variables in retired Rugby League players only (*n* = 41).

	**Abnormal CSP (*****n*** **= 25)**				**Normal CSP (*****n*** **= 16)**						
	**M**	**Md**	**SD**	**IQR**	**M**	**Md**	**SD**	**IQR**	**U**	***p***	***r***
Age	50.60	47.00	15.20	39.50–59.50	52.19	51.50	12.34	41.50–62.25	181.00	0.63	−0.08
Number of self-reported concussions	43.24	20.00	45.47	7.00–80.50	28.69	17.00	39.13	5.25–31.75	235.50	0.35	0.15
Age of first exposure to rugby league	8.28	6.00	4.21	5.50–10.00	7.56	7.00	3.52	4.25–9.75	213.50	0.72	0.06
Years played professionally	8.24	9.00	4.68	3.00–12.00	8.88	10.50	6.05	2.25–13.00	193.00	0.86	−0.03
Lifetime exposure to rugby league (Years)	23.56	25.00	5.61	20.50–28.00	25.06	26.00	6.09	22.25–28.50	174.50	0.50	−0.11
Perceived cognitive decline (IQCODE-self)^*^	3.22	3.19	0.45	2.94–3.50	3.22	3.19	0.47	3.00–3.46	123.50	0.73	−0.06
Depression (DASS)	5.60	4.00	8.29	0.00–10.00	8.25	7.00	7.04	0.50–15.50	143.50	0.13	−0.23
Daytime sleepiness (ESS)	6.04	4.00	5.05	2.50–11.00	4.63	4.00	3.93	1.25–8.25	225.00	0.52	0.11
Impulsivity (BIS)^†^	62.48	61.00	14.23	50.00–74.00	64.63	62.50	9.93	57.25–75.50	164.00	0.58	0.09
Estimated intelligence (ToPF reading)	102.16	102.00	11.10	95.00–111.50	100.94	101.00	8.98	93.25–107.75	215.00	0.70	0.06
Cognitive composite	48.80	49.33	6.16	44.67–52.79	47.09	47.25	3.55	44.52–50.25	252.50	0.16	0.22
RAVLT learning	40.44	42.00	10.31	34.50–48.50	41.88	42.00	8.57	35.00–45.75	191.50	0.82	0.04
RCFT immediate recall	43.60	45.00	9.31	37.50–50.00	38.63	37.50	7.61	33.25–43.50	271.50	0.06	0.30
RCFT delayed recall	44.24	45.00	8.42	39.50–50.50	40.25	39.00	7.67	35.25–45.75	265.50	0.08	0.27
Trails A	47.44	47.00	10.27	40.50–56.50	46.00	44.00	8.20	41.25–52.75	218.00	0.64	0.08
Trails B	56.40	58.00	12.18	52.00–64.50	54.13	56.50	8.43	46.00–60.50	241.00	0.28	0.17
Stroop inhibition	52.76	53.00	13.90	39.50–62.00	49.63	49.50	11.00	41.75–54.50	234.50	0.36	0.14
Phonemic fluency (COWAT FAS)	46.88	45.00	13.53	41.00–52.50	48.81	48.50	10.08	40.00–54.00	184.50	0.68	−0.06
Semantic fluency (animals)	56.80	59.00	13.96	46.00–67.00	50.38	53.00	10.42	41.75–59.75	258.50	0.12	0.24
WAIS-IV digit span backwards	47.92	47.00	8.74	41.50–53.00	46.50	47.00	5.51	43.00–50.00	205.00	0.91	0.02
WAIS-IV digit span sequencing	50.16	50.00	9.09	45.00–57.00	50.50	51.50	8.35	41.75–57.00	201.50	0.97	0.01
WAIS-IV coding	50.04	53.00	9.02	43.00–55.00	49.38	50.00	7.22	47.00–53.00	231.00	0.42	0.13
WAIS-IV symbol search	48.92	53.00	10.21	40.00–57.00	49.06	50.00	8.25	43.00–53.00	211.00	0.78	0.05

*CSP, Cavum Septum Pellucidum; IQR, Interquartile Range; Md, Median; M, Mean; SD, Standard Deviation; U, Mann Whitney U statistic; BIS, Barrett Impulsivity Scale; COWAT, Controlled Oral Word Association Test; DASS, Depression, Anxiety, Stress Scales; ESS, Epworth Sleepiness Scale; IQCODE-Self, Informant Questionnaire on Cognitive Decline in the Elderly, self-report form; RAVLT, Rey Auditory Verbal Learning Test; RCFT, Rey Complex Figure Test; ToPF, Test of Premorbid Functioning Reading Standard Score; WAIS-IV, Wechsler Adult Intelligence Scale-4th Edition*.

* *For Perceived Cognitive Decline, data were missing for two six participants with Abnormal CSP (n = 19) and for two participants with Normal CSP group (n = 14)*.

† *For Impulsivity, data were missing for two participants with Abnormal CSP, leading to a group size of 23 (total sample size = 39) for that variable. Effect sizes are interpreted as 0.1 = small, 0.3 = moderate, 0.5 = large*.

Clinical-anatomical associations were examined in the retired Rugby League players (*n* = 41), including measures of age, concussion exposure, and clinical variables. For the CSP/septal ratio, none of the 23 correlations were statistically significant. The Evans' ratio was positively associated with age (*r* = 0.48, *p* = 0.002) and perceived cognitive decline (IQ-CODE-Self; *r* = 0.39, *p* = 0.02), but none of the other Evans' ratio correlations were significant (Table 3).

**Table 3:** Spearman bivariate clinical-anatomic correlations in retired Rugby League players (*n* = 41).

	**CSP/septal ratio (Rater 1, PS)**	**Evans' ratio (Rater 1, PS)**
Age	*r* = −0.26, *p* = 0.11	***r =*** **0.48**, ***p*** **= 0.002**
Number of self-reported concussions	*r* = 0.18, *p* = 0.26	*r* = −0.13, *p* = 0.43
Age of first exposure to Rugby League	*r* = −0.10*, p* = 0.54	*r* = 0.01, *p* = 0.97
Years played professionally	*r* = 0.26, *p* = 0.10	*r* = −0.08, *p* = 0.62
Lifetime exposure to rugby league (years)	*r* = 0.07, *p* = 0.68	*r* = 0.12, *p* = 0.45
Perceived cognitive decline (IQCODE-self)^*^	*r* = 0.04. *p* = 0.82	***r*** **= 0.39*****, p*** **= 0.02**
Depression (DASS)	*r* = −0.17, *p* = 0.30	*r* = −0.001, *p* = 0.995
Daytime sleepiness (ESS)	*r* = 0.03, *p* = 0.88	*r* = 0.24, *p* = 0.13
Impulsivity (BIS)^†^	*r* = 0.05, *p* = 0.78	*r* = −0.09, *p* = 0.59
Estimated intelligence (ToPF reading)	*r* = 0.08, *p* = 0.62	*r* = 0.20, *p* = 0.22
Cognitive composite	*r* = 0.14, *p* = 0.37	*r* = −0.08, *p* = 0.61
RAVLT learning	*r* = 0.02, *p* = 0.88	*r* = −0.11, *p* = 0.50
RCFT immediate recall	*r* = 0.29, *p* = 0.07	*r* = −0.22, *p* = 0.17
RCFT delayed recall	*r* = 0.25, *p* = 0.11	*r* = −0.18, *p* = 0.25
Trails A	*r* = 0.05, *p* = 0.75	*r* = −0.22, *p* = 0.16
Trails B	*r* = 0.15, *p* = 0.36	*r* = −0.03, *p* = 0.88
Stroop inhibition	*r* = 0.16, *p* = 0.33	*r* = 0.04, *p* = 0.81
Phonemic fluency (COWAT FAS)	*r* = −0.05, *p* = 0.77	*r* = −0.05, *p* = 0.74
Semantic fluency (animals)	*r* = 0.17, *p* = 0.30	*r* = −0.04, *p* = 0.79
WAIS-IV digit span backwards	*r* = −0.16, *p* = 0.31	*r* = 0.23, *p* = 0.14
WAIS-IV digit span sequencing	*r* = −0.10, *p* = 0.53	*r* = 0.30, *p* = 0.06
WAIS-IV coding	*r* = 0.14, *p* = 0.38	*r* = −0.22, *p* = 0.16
WAIS-IV symbol search	*r* = 0.02, *p* = 0.90	*r* = −0.20, *p* = 0.20

*CSP, Cavum Septum Pellucidum; COWAT, Controlled Oral Word Association Test; DASS, Depression, Anxiety, Stress Scales; ESS, Epworth Sleepiness Scale; IQCODE-Self, Informant Questionnaire on Cognitive Decline in the Elderly, self-report form; RAVLT, Rey Auditory Verbal Learning Test; RCFT, Rey Complex Figure Test; ToPF, Test of Premorbid Functioning Reading Standard Score; WAIS-IV, Wechsler Adult Intelligence Scale-4th Edition*.

* *For Perceived Cognitive Decline, data were missing for eight participants, leading to a total sample size of 33 for these correlations*.

† *For Impulsivity, data were missing for two participants, leading to a total sample size of 39 for these correlations*.

## Discussion

The presence of a CSP is considered a normal anatomic variant sporadically seen on contemporary neuroimaging (16, 17). However, the presence of enlarged CSP on MRI in the general population is much less common (17). In a meta-analysis of 25 studies of CSP using MRI, CSP was identified in 55.9% of people with mood disorders or schizophrenia spectrum disorders compared to 50.0% of psychiatrically healthy control subjects, and a large CSP was found in 10.7% of those with mental disorders compared to 5.8% of control subjects (8). See Table 4 for a summary of these studies. There is considerable variability in the percentages of healthy control subjects with CSP, with 7/24 studies reporting fewer than 20% and 9/24 studies reporting 75% or greater.

**Table 4:** CSP in people with mental disorder and healthy control subjects, visible on MRI.

	**People with** **mental disorders**			**Psychiatrically** **healthy** **control subjects**			**People with mental disorders**			**Psychiatrically healthy control subjects**		
**References**	**CSP**	**Total**	**%** **patients**	**CSP**	**Total**	**%** **controls**	**Large CSP**	**Total**	**%** **patients**	**Large CSP**	**Total**	**%** **controls**
Degreef et al. (62)	22	62	35.5	7	46	15.2	–	–	–	–	–	–
Jurjus et al. (63)	23	127	18.1	7	37	18.9	–	–	–	–	–	–
DeLisi et al. (64)	38	85	44.7	14	47	29.8	–	–	–	–	–	–
Myslobodsky et al. (10)	5	10	50.0	0	10	0.0	–	–	–	–	–	–
Shioiri et al. (65)	12	153	7.8	1	92	1.1	–	–	–	–	–	–
Nopoulos et al. (66)	32	55	58.2	44	75	58.7	6	55	10.9	1	75	1.3
Fukuzako and Kodama (67)	34	72	47.2	16	41	39.0	–	–	–	–	–	–
Kwon et al. (68)	49	67	73.1	39	46	84.8	12	67	17.9	4	46	8.7
Rajarethinam et al. (69)	44	73	60.3	18	43	41.9	3	73	4.1	1	43	2.3
Keshavan et al. (70)	4	40	10.0	7	59	11.9	–	–	–	–	–	–
Galarza et al. (71)	14	32	43.8	2	19	10.5	–	–	–	–	–	–
Crippa et al. (72)	16	21	76.2	18	21	85.7	0	21	0.0	0	21	0.0
Kasai et al. (73)	56	74	75.7	49	56	87.5	–	–	–	–	–	–
de Souza Crippa et al. (74)	30	38	78.9	33	38	86.8	8	38	21.1	1	38	2.6
Borgwardt et al. (75)	1	30	3.3	0	26	0.0	–	–	–	–	–	–
Kim et al. (7)	28	41	68.3	21	41	51.2	8	41	19.5	1	41	2.4
Flashman et al. (42)	53	77	68.8	42	55	76.4	11	77	14.3	5	55	9.1
Takahashi et al. (76)	157	201	78.1	133	163	81.6	15	201	7.5	12	163	7.4
Takahashi et al. (77)	223	251	88.8	78	87	89.7	–	–	–	–	–	–
Takahashi et al. (78)	48	56	85.7	28	33	84.8	3	56	5.4	5	33	15.2
Chon et al. (79)	43	71	60.6	21	71	29.6	5	71	7.0	3	71	4.2
Takahashi et al. (80)	22	26	84.6	18	24	75.0	–	–	–	–	–	–
Hwang et al. (81)	54	65	83.1	43	67	64.2	12	65	18.5	4	67	6.0
Toivonen et al. (82)	–	–	–	–	–	–	2	26	7.7	2	25	8.0
Landin-Romero et al. (11)	314	639	49.1	71	223	31.8	–	–	–	–	–	–
Totals	1,322	2,366	55.9%	710	1,420	50.0%	85	791	10.7%	39	678	5.8%

*The sample sizes for each study were derived from Table 1 in Wang et al. (8). The percentages were not included in that article and were computed for this article. There was on error in the original Table 1 in the published article, where the sample size in Chon et al., in two places, was listed as 77 instead of 71. CSP, Cavum Septum Pellucidum; %, percentage. A large CSP was defined as ≥6 mm in length. Those with mental disorders had schizophrenia spectrum disorders or mood disorders*.

Enlarged CSP on MRI has been reported in some retired American professional football players (27–29), boxers (24, 25), and in a mixed sample of combat sport athletes (83). Moreover, Aviv and colleagues (24) identified a high percentage of CSP in both boxers (81/164; 49%) and control subjects (17/43; 40%) in a study of British professional boxers undergoing annual MRI to maintain registration, with it being a little more common in boxers. The majority of boxers had a single study (52%) or two studies (33.5%), while 24 boxers had >3 scans. Three boxers increased the extent of CSP over serial MRI studies, while eight boxers demonstrated a CSP on a subsequent scan not seen on an earlier scan indicating the CSP may be an acquired abnormality, and may change in size over time (24). There was no difference in CSP size between boxers and controls, but there was an association between progressive scans and increased CSP size over time in boxers. However, no control subjects were imaged at more than one time point, additionally there was an absence of information regarding exposure to repetitive neurotrauma (duration of boxing, number of bouts, knockouts, concomitant head injury). The Evans' index was also studied with no difference between boxers compared to control participants.

Gardner and colleagues compared 17 retired NFL players to 17 control participants and found that 16/17 former players (94%) had some degree of CSP (grade ≥ 2) compared to 3/17 controls (18%) (28). In a study of 45 retired NFL players, a small CSP was common in the retired players (32/45; 71%), and a few had a large CSP (3/45; 7%), but CSP was not significantly correlated with any measure of self-reported symptoms or neuropsychological test scores (27). Koerte et al. (29) compared 72 symptomatic retired NFL players to 14 control participants and found that the retired players had a greater ratio of CSP length to septal length. They also divided the retired players into two groups and compared them on 24 measures of self-reported mental health, neurobehavioral symptoms, and neuropsychological test results. Significant differences were found on only two measures, a test of verbal learning (immediate recall) and a test of word reading. Similarly, in the present study, there were no significant differences on 18/18 clinical measures (Table 2) between retired players stratified on CSP. We did not replicate their two significant findings relating to verbal learning (RAVLT) or reading (TOPF reading score).

The present study revealed that retired elite level rugby league players, on average, had a significantly larger CSP, with a moderate effect size, than the control participants (Table 1). There was a 20% difference in the proportions of retired players who have an *abnormal* CSP (61.0%) compared to control participants (41.5%), but the difference was not statistically significant which could reflect a type II statistical error given our relatively small sample sizes. No individual in either group had a “severe” CSP (rating of 4/4), and very few in either group were rated as having a “moderate” CSP (i.e., 1/41, 2.4%, controls; and 4/41, 9.8% retired players). The vast majority of those in both groups who were rated as having a CSP had a “mild” CSP. There was no significant difference in the size of the lateral ventricles between retired players and control participants (very small effect size, Table 1), suggesting that retired players have not experienced obvious global atrophy that might underlie volumetric changes in the ventricles. Thus, there were relatively small differences in CSP and no difference in ventricular size between the groups.

Importantly, in the present study, there were no significant clinical correlations with CSP. Within the retired players group, there were no significant differences between those with vs. without an abnormal CSP and their lifetime exposure to repetitive neurotrauma, measured by the age of their first exposure to rugby league (84), total number of years of exposure, number of years of elite (professional) play, or number of lifetime concussions. Moreover, there were no significant differences between retired players with vs. without a CSP on measures of depression, impulsivity, perceived cognitive decline, or on any neuropsychological test.

As expected, there was a significant correlation between age and ventricle size, in both groups, consistent with ventricular enlargement occurring with aging (40, 85–89). There was also a small significant positive correlation between the Evans' ratio and the IQCODE in the retired players, indicating an association between larger ventricles, suggestive of cerebral atrophy, and greater perceived cognitive decline. This finding adds to a mixed literature on the relationship between perceived (or “subjective”) cognitive decline and structural neuroimaging findings such as atrophy and ventricular asymmetry (90, 91). Importantly, aspects of the individual participant samples (92), as well as the measurement and definition of perceived cognitive decline (93), likely contribute to this variation in the literature.

Lastly, there was a small significant negative correlation between the size of the CSP and the size of the ventricles in the control participants, indicating that larger ventricles are associated with a smaller CSP (for unknown reasons). There was no correlation between the size of the CSP and the size of the ventricles in the retired players, and retired players with an abnormal CSP did not have larger ventricles than retired players who did not have an abnormal CSP.

### Limitations

There are several limitations of the current study. Most importantly, it is not possible to claim that the sample is representative of all retired elite rugby league players because it represents those who volunteered to participate in this extensive brain health research program. Our sample may not fully represent either end of the cognitive health spectrum, from those who are the healthiest and highest functioning, to those who are the most neurologically compromised. Second, the study design is cross-sectional and, therefore, we are unable to infer causality or exclude the possibility that CSP was present developmentally in some retired rugby league players. Third, concussion history and contact sport exposure were measured retrospectively, via self-report, and this method is vulnerable to recall failures and biases. Finally, in comparison with some other CSP studies in retired athletes, our sample size was smaller.

## Conclusions

In the present study, the presence of a CSP was a common finding in both groups. Consistent with the primary hypothesis, there was a statistically significant difference in the size of CSP between retired players and controls (i.e., the CSP/septal ratio). Contrary to our secondary hypothesis, there was no significant difference in the Evans' ratio between retired players and control participants, suggesting no difference in the size of lateral ventricles and no evidence of obvious global cerebral atrophy. Moreover, within the retired players group, there was no significant difference in the size of the lateral ventricles between those with an abnormal CSP and those without a CSP. Importantly, there was no association between the presence or size of the CSP and age of first exposure to collision sport, total number of years playing the sport, number of years playing at the elite (professional) level, lifetime history of concussions, depression, impulsivity, perceived decline in cognitive functioning, or a battery of neuropsychological tests. One interpretation of larger CSPs in retired collision sport athletes compared to controls is that repetitive neurotrauma leads to increased CSP size. However, the present study, and prior studies, suggest the clinical significance of enlarged CSP in retired contact and collision sport athletes remains unclear.

## Data Availability Statement

The datasets presented in this article are not readily available because the study is ongoing and the MRI scans are not being uploaded into a repository. The statistical analyses and outputs are available to qualified researchers, for research purposes. Requests to access this information can be made to Dr. Andrew Gardner (Andrew.Gardner@newcastle.edu.au).

## Ethics Statement

The studies involving human participants were reviewed and approved by University of Newcastle. The patients/participants provided their written informed consent to participate in this study.

## Author Contributions

PS conceptualized the study, assisted with the literature review, conceptualized and conducted the MRI analyses, and wrote portions of the manuscript. GI helped conceptualize the study, assisted with the literature review, helped conceptualize and conduct the statistical analyses, wrote portions of the manuscript, and secured funding for the study. RV assisted with the literature review, helped conceptualize and conduct the statistical analyses, wrote portions of the manuscript, and critically reviewed drafts of the manuscript. RC and PM assisted with the literature review and critically reviewed drafts of the manuscript. AG is the principal investigator for the research program with retired rugby league players, he designed and manages the overall study, conducted all of neuropsychological evaluations, designed and constructed the database, helped conceptualize the study, conducted the MRI analyses as a second rater, assisted with the literature review, and critically reviewed drafts of the manuscript. All authors read and approved the last version of this manuscript.

## Funding

This study received funding, in part, from the National Football League for a program of research entitled The Spectrum of Concussion: Predictors of Clinical Recovery, Treatment and Rehabilitation, and Possible Long-Term Effects (PI GI). PS was funded by the Australian-American Fulbright Commission. The funders were not involved in the study design, collection, analysis, interpretation of data, the writing of this article or the decision to submit it for publication. In addition, unrestricted philanthropic support was provided by ImPACT Applications, Inc., the Mooney-Reed Charitable Foundation, the National Rugby League, and the Spaulding Research Institute. Those entities were not involved in the study design, collection, analysis, interpretation of data, the writing of this article or the decision to submit it for publication.

## Conflict of Interest

PS has received previous grant funding from the NSW Sporting Injuries Committee, the Brain Foundation (Australia), and the Australian-American Fulbright Commission. GI serves as a scientific advisor for NanoDx™, Sway Operations, LLC, and Highmark, Inc. He has a clinical and consulting practice in forensic neuropsychology, including expert testimony, involving individuals who have sustained mild TBIs (including athletes). He has received research funding from several test publishing companies, including ImPACT Applications, Inc., CNS Vital Signs, and Psychological Assessment Resources (PAR, Inc.). He has received research funding as a principal investigator from the National Football League, and salary support as a collaborator from the Harvard Integrated Program to Protect and Improve the Health of National Football League Players Association Members. RC has received research funding as principal investigator from the Chuck Knoll Foundation. He has provided expert testimony involving deceased individuals who have sustained traumatic brain injury and deceased former athletes. PM is a co-investigator on competitive grants relating to mild TBI funded by several governmental and other organizations. He is funded under a Fellowship awarded by the National Health & Medical Research Council of Australia and the Medical Research Future Foundation of Australia. He is employed at the Florey Institute of Neuroscience and Mental Health. He has a clinical consulting practice in neurology, including medico-legal work. He has been reimbursed by the government, professional scientific bodies, and commercial organizations for discussing or presenting research relating to MTBI and sport-related concussion at meetings, scientific conferences, and symposiums. He does not hold any individual shares in or receive monies from any company related to concussion or brain injury assessment or technology. He acknowledges unrestricted philanthropic support from CogState Inc. (2001-16). He is the chair of the scientific committees of the International Concussion and Head Injury Research Foundation in London and the Sports Surgery Clinic in Dublin. AG serves as a scientific advisor for HitIQ, Ltd. He has a clinical practice in neuropsychology involving individuals who have sustained sport-related concussion (including current and former athletes). He has been a contracted concussion consultant to Rugby Australia. He is a member of the World Rugby concussion working group. He has received travel funding or been reimbursed by professional sporting bodies, and commercial organizations for discussing or presenting sport-related concussion research at meetings, scientific conferences, workshops, and symposiums. Previous grant funding includes the NSW Sporting Injuries Committee, the Brain Foundation (Australia), an Australian-American Fulbright Commission Postdoctoral Award, an NHMRC Early Career Fellowship, a Hunter New England Local Health District, Research, Innovation and Partnerships Health Research & Translation Centre and Clinical Research Fellowship Scheme, and the Hunter Medical Research Institute (HMRI), supported by Jennie Thomas, and the HMRI, supported by Anne Greaves. He is currently funded through the University of Newcastle's Priority Research Centre for Stroke and Brain Injury. He has received research funding from the National Rugby League (NRL) and the Australian Institute of Sport (AIS) for the Retired Players Brain Health research program. The remaining authors declare that the research was conducted in the absence of any commercial or financial relationships that could be construed as a potential conflict of interest.

## Publisher's Note

All claims expressed in this article are solely those of the authors and do not necessarily represent those of their affiliated organizations, or those of the publisher, the editors and the reviewers. Any product that may be evaluated in this article, or claim that may be made by its manufacturer, is not guaranteed or endorsed by the publisher.
